# Metabolic Profiling of Total Physical Activity and Sedentary Behavior in Community-Dwelling Men

**DOI:** 10.1371/journal.pone.0164877

**Published:** 2016-10-14

**Authors:** Kota Fukai, Sei Harada, Miho Iida, Ayako Kurihara, Ayano Takeuchi, Kazuyo Kuwabara, Daisuke Sugiyama, Tomonori Okamura, Miki Akiyama, Yuji Nishiwaki, Yuko Oguma, Asako Suzuki, Chizuru Suzuki, Akiyoshi Hirayama, Masahiro Sugimoto, Tomoyoshi Soga, Masaru Tomita, Toru Takebayashi

**Affiliations:** 1 Department of Preventive Medicine and Public Health, Keio University School of Medicine, Tokyo, Japan; 2 Institute for Advanced Biosciences, Keio University, Tsuruoka, Japan; 3 Department of Obstetrics and Gynecology, Keio University School of Medicine, Tokyo, Japan; 4 Faculty of Environment and Information Studies, Keio University, Fujisawa, Japan; 5 Division of Environmental and Occupational Health, Department of Social Medicine, Faculty of Medicine, Toho University, Tokyo, Japan; 6 Sports Medicine Research Center, Keio University, Yokohama, Japan; 7 Graduate School of Health Management, Keio University, Fujisawa, Japan; National Research Council of Italy, ITALY

## Abstract

**Objective:**

Physical activity is known to be preventive against various non-communicable diseases. We investigated the relationship between daily physical activity level and plasma metabolites using a targeted metabolomics approach in a population-based study.

**Methods:**

A total of 1,193 participants (male, aged 35 to 74 years) with fasting blood samples were selected from the baseline survey of a cohort study. Information on daily total physical activity, classified into four levels by quartile of metabolic equivalent scores, and sedentary behavior, defined as hours of sitting per day, was collected through a self-administered questionnaire. Plasma metabolite concentrations were quantified by capillary electrophoresis mass spectrometry method. We performed linear regression analysis models with multivariable adjustment and corrected p-values for multiple testing in the original population (n = 808). The robustness of the results was confirmed by replication analysis in a separate population (n = 385) created by random allocation.

**Results:**

Higher levels of total physical activity were associated with various metabolite concentrations, including lower concentrations of amino acids and their derivatives, and higher concentrations of pipecolate (FDR p <0.05 in original population). The findings persisted after adjustment for age, body mass index, smoking, alcohol intake, and energy intake. Isoleucine, leucine, valine, 4-methyl-2-oxoisopentanoate, 2-oxoisopentanoate, alanine, and proline concentrations were lower with a shorter sitting time.

**Conclusions:**

Physical activity is related to various plasma metabolites, including known biomarkers for future insulin resistance or type 2 diabetes. These metabolites might potentially play a key role in the protective effects of higher physical activity and/or less sedentary behavior on non-communicable diseases.

## Introduction

A large body of evidence has documented the beneficial effects of physical activity on the prevention of non-communicable diseases [1–3]. Epidemiological studies of total physical activity (TPA) related to daily living and work have reported dose-dependent health benefits, such as reductions in all-cause mortality, fatal and non-fatal cardiovascular disease, type 2 diabetes (T2D), and metabolic syndrome [4–7]. However, the underlying mechanisms by which TPA exerts these various health benefits are poorly understood [8].

Metabolomics is the comprehensive analysis of metabolites in a biologic sample. This provides a functional assessment of physiologic processes and is ideally suited to describing individual health status [9–11]. Metabolic profiling using metabolomics in epidemiological studies allows us to comprehensively identify metabolic alterations in relation to lifestyle factors, such as physical activity, and to indicate potential biomarkers related to metabolic diseases [12–14]. For example, Wang et al. [15] reported plasma metabolite levels of branched-chain and aromatic amino acids as predictors of future T2D. To date, however, only a few studies have described metabolic alterations from habitual, daily physical activities in an epidemiological setting. Kujala et al. [13] reported associations of physical activity with various metabolites, including isoleucine, glucose, glycoprotein, and lipids, while Wientzek et al. [16] showed an association with phosphatidylcholines. However, although race differences in metabolic profiles have been reported [17], no study has reported associations between metabolic profiling and daily physical activity in an Asian population, which are generally lean and have different dietary habits from Westen populations.

Here, conducted an epidemiological study in a Japanese population by profiling metabolic alterations in relation to TPA levels using capillary electrophoresis mass spectrometry (CE-MS), a method which provides high-resolution, quantitative metabolome analysis for polar metabolites, including amino acids and carbohydrates [18,19]. Further, given longitudinal findings over the last decade of the adverse health effects of excessive time in sedentary behaviors, such as sitting at work or using computers, regardless of levels of physical activity [20–23], we also evaluated the effects of sedentary behavior.

## Material and Methods

### Study population

The study was conducted with baseline survey participants of the Tsuruoka Metabolomics Cohort Study, a population-based study started in April 2012 among residents aged 35–74 years of Tsuruoka City, Yamagata Prefecture, Japan. The participants were recruited from a municipal health checkup program during the recruiting period of this study. Details have been reported previously [14,24]. For the present study, 1,232 men (all participants in the first six months) whose plasma metabolome analysis was completed were subject to analysis. Among study subjects, we excluded those with incomplete (n = 30) or inaccurate (n = 1) physical activity data, inability to walk 50 meters due to a physical disability (n = 8), morning exercise before the blood collection (n = 6), and non-fasting blood samples (n = 1). We also excluded a subject whose metabolic profile had been detected as an outlier in the primary component analysis (n = 1; PC1 score of +43.4, versus range of -8.6 to +9.1).

Since replicability is important in confirming study results, we divided the subjects into two groups (2:1) using random stratification by age-class (less than 65 years, 65 years or more) and measurement batch. The final number of subjects was 808 men in the original, explanatory dataset and 385 men in the replication dataset. Participant flow is outlined in S1 Fig.

The study protocol was approved by the Medical Ethics Committee of the School of Medicine, Keio University, Tokyo, Japan (approval no. 20110264). All participants in the study provided written informed consent.

### Data and sample collection

All data were collected during the annual health check-up. The participants responded to a self-administered questionnaire which included information on physical activity, demographic factors, alcohol drinking, smoking habit, personal medical history, and other lifestyle factors. Daily dietary energy intake was assessed based on a validated short food frequency questionnaire, comprising 47 food items [24,25]. Other measurements included height and weight to calculate body mass index (BMI), blood pressure, and routine blood tests conducted in the check-up, including serum triglyceride, cholesterols, hemoglobin A1c, and plasma glucose. Hypertension was defined as systolic blood pressure ≥ 140 mmHg and/or diastolic blood pressure ≥ 90 mmHg and/or currently on antihypertensive therapy; impaired glucose tolerance as fasting plasma glucose ≥ 110mg mg/dL and/or hemoglobin A1c (NGSP) ≥ 6.5% and/or current use of antidiabetic medication; and dyslipidemia as serum triglyceride ≥ 150 mg/dL and/or low-density lipoprotein cholesterol ≥ 140 mg/dL and/or high-density lipoprotein cholesterol ≤ 40 mg/dL and/or current use of antidyslipidemic medication.

### Physical activity assessment

In the questionnaire, subjects were asked about the average time spent in six types of physical activity during the last 12 months, namely daily heavy physical work, daily walking, daily sitting, leisure-time very vigorous physical activity (*e*.*g*. running, fighting sports), leisure-time vigorous physical activity (*e*.*g*. jogging, skiing), and leisure-time moderate physical activity (*e*.*g*. walking the dog, walking for pleasure, home exercise). For daily activities, participants were asked about average time spent per day (none, <30 min, 30 min- <1 h, 1 h- <3 h, 3 h- <5 h, 5 h- <7 h, 7 h- <9 h, 9 h- <11 h, or 11 h≤); and for leisure-time activities, about duration per time (none, <30 min, 30 min- <1 h, 1 h- <2 h, 2 h- <3 h, 3 h- <4 h, or 4 h≤) and frequency (none, once per month, 2–3 times per month, 1–2 times per week, 3–4 times per week, 5≤ times per week). The intensity of each activity was assigned using the Ainsworth compendium of physical activities as follows: 4.5 Metabolic equivalents (METs) for daily heavy physical work, 3.0 METs for daily walking, 10.0 METs for leisure-time very vigorous physical activity, 7.0 METs for leisure-time vigorous physical activity, and 3.4 METs for leisure-time moderate physical activity [26]. The total amount of physical activity per week (TPA, MET-hours/week) was calculated by multiplying the intensity of each type of physical activity, time spent in the activity, and frequency of leisure-time physical activity according to the questionnaire. The midpoint of the time range and frequency for each category were used to calculate MET-hours/week [27].

Sedentary behavior was assessed by the questionnaire with the following question: “During a usual day and night, how many hours did you spend sitting this past year on average? Be sure to include the time you spent sitting at work, home, leisure time, and during transportation.” Nine categories were provided for the response as provided above.

The validity of the total MET-hours/week score and sitting time/day was assessed among 53 male volunteers among the cohort using a triaxial accelerometer (Active style Pro, Omron HealthCare, Kyoto, Japan) [28,29]. Participants were asked to wear the accelerometer the whole time except when they were bathing or in bed for 7 consecutive days, and samples from subjects who wore the device for 10 h/day or more for at least 4 days, were analyzed. Spearman’s rank correlation coefficient for the correlation between total MET score measured by the accelerometer and the questionnaire was 0.37, while that of sitting time was 0.44, which were similar to previous findings [30,31].

### Metabolomics measurement

Blood samples were collected in the morning between 8:30 and 10:30 after a ≥12 h overnight fast to avoid variation due to fasting state. Targeted metabolite profiling was performed with plasma samples via capillary electrophoresis time-of-flight mass spectrometry (CE-TOFMS). To minimize the effect of metabolic change prior to sample processing, plasma samples were kept at 4°C right after blood drawing, and metabolite extraction was completed within six hours after collection. Samples were then kept at -80°C until analyzed by the CE-TOFMS metabolomics platform. These protocols, including sample preparation and CE-TOFMS methods, have been described in detail elsewhere [32]. This platform provides quantitative and high-resolution metabolome analysis for cationic and anionic metabolites. The raw data were processed using our proprietary software (MasterHands) [18]. In this study, we routinely conducted measurement of absolute concentrations of 115 metabolites (63 cations and 52 anions) that were expected to be stably observed in most human plasma samples and had matched standards [14].

### Statistical analysis

Metabolite concentrations were treated as continuous variables and log-transformed. For metabolites data, we excluded metabolites which had plasma concentration levels below the assay limits of detection (LOD) in more than 90% of the whole population, and selected 77 metabolite concentrations for further analysis. For samples with undetectable levels below the LOD, their values were imputed using half of the LOD values [33]. We also conducted the same analysis for routine clinical chemistry items.

TPA level (calculated as MET-hours/week for each subject) was classified by quartile (Q1-Q4). First, to examine the association between plasma metabolite concentrations and TPA, simple linear regression analysis was performed in the original population to elucidate the effect of a one-level increase in TPA (1–4 as Q1-Q4) on metabolite concentrations. To adjust multiple testing corrections, we presented p values using Benjamini and Hochberg’s false discovery rate (FDR) method [34]. Age, body mass index (BMI), smoking (never/former/current), alcohol intake (none/current), daily dietary energy intake without alcohol (higher/lower than median) were then included as potential confounders for adjusted analysis. Replication analysis was done by simple and multivariable linear regression analysis for metabolites that were statistically associated with TPA levels for the unadjusted model in the original population (FDR p <0.05).

Daily sitting time as an index of sedentary behavior was categorized into three groups, namely short (<3 h), medium (3- <7 h), and long (≥7 h), in accordance with previous studies [23,27]. We conducted the same analysis for sedentary behavior.

To further explore the combined effects of TPA and sedentary behavior, we calculated adjusted mean concentrations of metabolites which showed associations with both TPA and sedentary behavior in groups cross-classified by TPA level and daily sitting time level. Interaction terms were added to the regression model to test the interactions between TPA and daily sitting time.

Various effects on the circulating metabolome have been reported, including those due to cancer or the use of drugs [11,18,35]. Sensitivity analysis was performed by excluding participants who had a history of cancer, as well as those who were taking medication for hypertension, dyslipidemia or diabetes. SAS 9.3 (SAS Institute Inc., Cary, NC) was used for all statistical analysis.

## Results

### Characteristics

Table 1 shows the characteristics of study subjects by quartile of TPA level. Median total scores for the TPA groups, namely Q1, Q2, Q3, Q4, were 24.0, 68.3, 131.4, and 273.0 MET-hours/week, respectively, in the original population, and 24.0, 70.3, 132.5, and 270.6, respectively, in the replication population (S1 Table). In the original population, participants in the Q4 group reported the highest alcohol intake and current smoking. Similar trends were observed in the replication population. HDL-cholesterol and triglyceride were associated with TPA levels. There were no significant differences in the history of coronary heart disease, cerebral vascular disorder, or cancer among TPA levels in either population.

**Table 1:** Characteristics of the original population (n = 808).

Characteristic	TPA-Q1 (n = 205)		Q2 (n = 210)		Q3 (n = 192)		Q4 (n = 201)		P-value^c^
Total physical activity (MET-hours/week)^a^	24.7	(0–43.7)	68.3	(44–96.2)	133.5	(96.5–191.6)	273.0	(192.8–630.2)	< 0.001
Age (years)^b^	62.3	(7.9)	62.9	(7.7)	63.4	(7.1)	62.2	(8)	0.457
BMI (kg/m^2^)^b^	23.8	(3.1)	23.7	(3.3)	23.8	(2.9)	23.0	(2.9)	0.032
Current smoking, Yes	23.9%	(49/205)	26.7%	(56/210)	25.5%	(49/192)	34.3%	(69/201)	0.022
Ex-smoker	55.6%	(114/205)	57.1%	(120/210)	54.2%	(104/192)	41.3%	(83/201)	-
Alcohol intake (g/day)^b^	28.6	(40.9)	28.3	(32.2)	27.4	(28.9)	34.2	(31.6)	0.004
Energy without alcohol (kcal/day)^b^	1790.5	(391.8)	1815.5	(504.1)	1889.0	(390.7)	1983.8	(448.6)	< 0.001
SBP (mmHg)^b^	131.4	(16.8)	130.7	(19.3)	132.7	(18.7)	129.9	(19.7)	0.325
DBP (mmHg)^b^	79.5	(10.1)	78.7	(10.7)	78.4	(10.4)	77.8	(11.7)	0.293
Hypertension^1^, Yes	52.7%	(108/205)	54.8%	(115/210)	54.2%	(104/192)	43.3%	(87/201)	0.072
Hypertension on medication	37.1%	(76/205)	37.6%	(79/210)	34.9%	(67/192)	26.4%	(53/201)	0.046
FPG (mg/dL)^a^	101.0	(80–175)	100.5	(83–200)	101.0	(81–211)	100.0	(76–230)	0.996
HbA1c (%)^a^	5.7	(4.9–8.2)	5.7	(4.8–10)	5.6	(5–9.4)	5.6	(4.9–9.3)	0.473
IGT^2^, Yes	29.8%	(61/205)	26.7%	(56/210)	26%	(50/192)	24.9%	(50/201)	0.714
IGT on medication	14.1%	(29/205)	9.5%	(20/210)	8.9%	(17/192)	8.5%	(17/201)	0.201
Triglyceride (mg/dL)^a^	127.6	(31–872)	135.8	(33–1879)	123.3	(32–565)	110.1	(37–431)	< 0.001
LDL cholesterol (mg/dL)^b^	114.9	(29.4)	116.6	(28.3)	119.3	(31.4)	114.0	(31.6)	0.177
HDL cholesterol (mg/dL)^b^	59.5	(14.9)	62.6	(14.3)	63.3	(15.6)	67.6	(17.2)	< 0.001
Dyslipidemia^3^, Yes	49.8%	(102/205)	54.3%	(114/210)	46.9%	(90/192)	37.3%	(75/201)	0.006
Dyslipidemia on medication	18.5%	(38/205)	15.2%	(32/210)	14.1%	(27/192)	8.0%	(16/201)	0.021
History of coronary heart disease	3.9%	(8/205)	3.3%	(7/210)	1.6%	(3/192)	0.5%	(1/201)	0.082
History of cerebral vascular disorder	5.4%	(11/205)	2.4%	(5/210)	6.3%	(12/192)	3.0%	(6/201)	0.182
History of cancer	3.9%	(8/205)	6.7%	(14/210)	4.7%	(9/192)	5.0%	(10/201)	0.585

^a^Reported as median (range).

^b^Reported as mean (standard deviation).

^c^P-values of the chi-sqare test for categorical variables, analysis of variance for continuous variables.

^1^Hypertension: systolic blood pressure ≥ 140 mmHg, diastolic blood pressure ≥ 90 mmHg, or on medication.

^2^Impaired glucose tolerance: FPG ≥ 110 mg/dL, hemoglobin A1c ≥ 6.5%, or on medication.

^3^Dyslipidemia: triglyceride ≥ 150 mg/dL, LDL cholesterol ≥ 140 mg/dL, HDL cholesterol ≤ 40 mg/dL, or on medication.

TPA, total physical activity; SBP, systolic blood pressure; DBP, diastolic blood pressure; FPG, fasting plasma glucose; IGT, impaired glucose tolerance; LDL, low-density lipoprotein; HDL, high-density lipoprotein.

### Association between TPA level and metabolome

**Fig 1:**
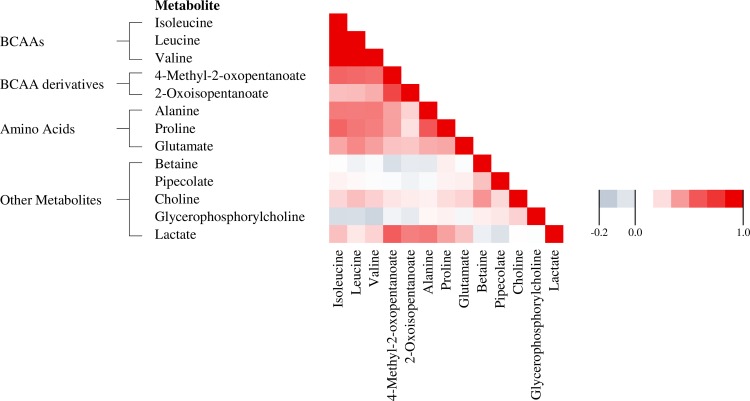
Correlation matrix for plasma metabolite levels: Pearson correlation coefficients for 13 metabolite concentrations (log-transferred) associated with TPA in the original dataset.

We first assessed the correlations between plasma metabolite concentrations (Fig 1). Mean correlation coefficient within groups of related metabolites was highest for the three branched-chain amino acids (BCAAs), *i*.*e*. isoleucine, leucine, and valine (age-adjusted *r* = 0.84).

**Fig 2:**
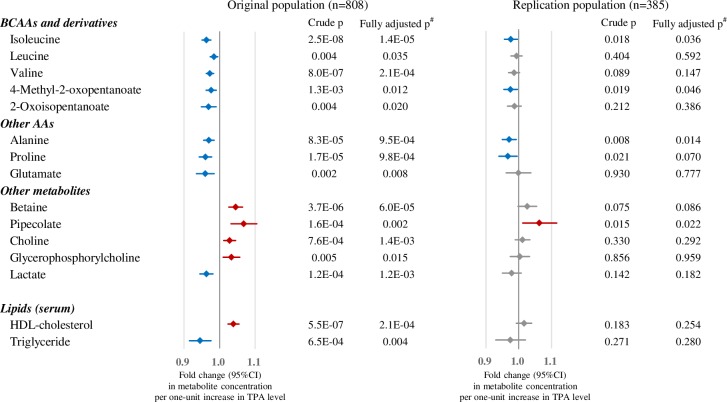
Associations between TPA and metabolite measurements. Associations between metabolites and TPA level (Q1, Q2, Q3, Q4) in the original (left) and replication (right) populations. Linear regression between each metabolite and TPA was performed. Raw p-values for unadjusted and adjusted models are shown. Fold change (with 95% CI) per one-unit increase in TPA level were calculated using the beta of the linear regression analysis of the unadjusted model. The blue bars mean lower concentrations and the red bars mean higher concentrations in highly active groups. Metabolites associated with TPA levels are shown in this figure (FDR p <0.05 for unadjusted model in the original population). Replication analysis was performed only for these metabolites. AAs, amino acids; CI, confidence interval. ^#^Adjusted for age, BMI, smoking (never/former/current), current alcohol drinker (yes/no), and energy intake (high/low).

Fig 2 shows the metabolites associated with TPA level. Concentrations of 13 plasma metabolites showed linear trends with relation to TPA level in the original dataset even after adjustment for multiplicity (FDR p <0.05), of which concentrations of five (isoleucine, 4-methyl-2-oxopentanoate, alanine, proline, and pipecolate) were replicated. Among these five metabolites, the concentrations of four (isoleucine, proline, alanine, and 4-methyl-2-oxopentanoate) were decreased in the higher active TPA group and were increased for pipecolate. The results were consistent with the inclusion or exclusion of various potential confounders, namely age, BMI, alcohol intake, smoking status, or dietary intake. In addition to our findings for these metabolites, our results also show the known associations of higher HDL-cholesterol and lower triglyceride concentration with physical activity [36]. Crude and fully-adjusted p-values are shown in Fig 2 (full data available in S2 Table).

### Association between sedentary behavior and metabolome

**Fig 3:**
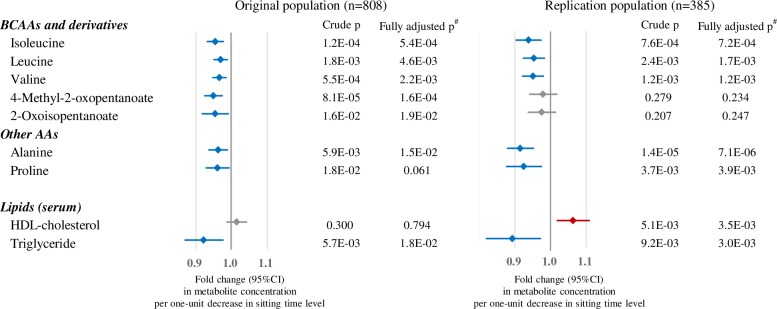
Associations between sedentary behavior level and metabolite measurements. Associations between metabolites and sitting time level (long, medium, short) in the original (left) and replication (right) populations. Linear regression between each metabolite was performed, and p-values for unadjusted and adjusted models are shown. Fold change per one-unit decrease with 95% CI in sitting time level were calculated using the beta of the linear regression analysis of the unadjusted model. Blue bars mean lower concentrations in the shorter sitting time groups. Only metabolites associated with sitting time levels (p <0.05 for the unadjusted model in the original population) among metabolites associated with TPA (shown in Fig 2) are shown. ^#^Adjusted for age, BMI, smoking (never/former/current), current alcohol drinker (yes/no), and energy intake (high/low).

The results of simple and multiple linear regression analyses in relation to daily sitting time level among the metabolites that were associated with TPA levels in the original dataset are shown in Fig 3 and S3 Table. Isoleucine, leucine, valine, alanine and proline showed a decrease with shorter sitting time in both the original and replication analysis. 4-Methyl-2-oxopentanoate and 2-oxoisopentanoate showed similar trends.

### Cross-classified multivariable-adjusted concentrations of key metabolites

**Fig 4:**
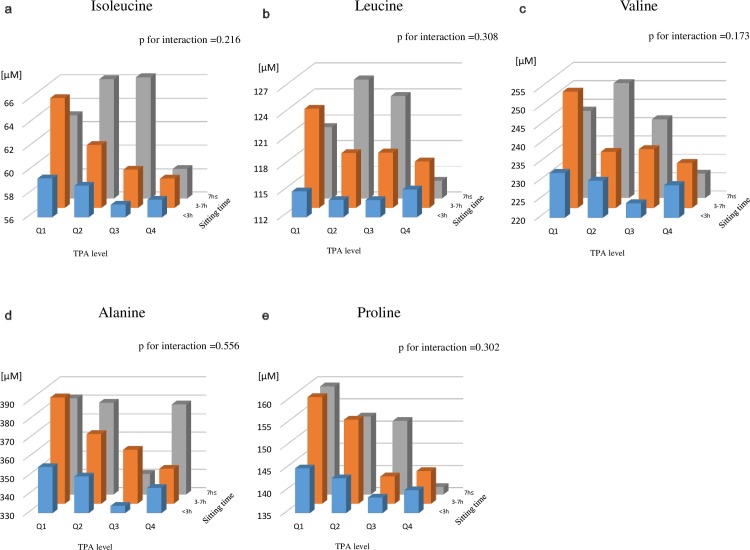
Cross-classified multivariable-adjusted mean concentrations of metabolites associated with TPA and sedentary behavior levels. Multivariable-adjusted (age, BMI, smoking, alcohol, energy intake) mean concentrations were calculated by groups cross-classified by TPA level and sitting time. Metabolites that showed a strong association with both exposures (Figs 2 and 3) are shown. P-values for interactions between TPA and sitting time levels were tested. Details of the number of subjects in each cross-classified group are shown in S2 Fig.

Fig 4 and S2 Fig show the multivariable-adjusted mean concentrations cross-classified by TPA and sedentary behavior levels for seven metabolites that were associated with both TPA and daily sitting time. Allowing that some sub-groups were small in number (*e*.*g*. 2% of the TPA-Q4 group reported sitting for ≥7 h/day), mean plasma concentrations of these metabolites decreased while both daily physical activity increased and daily sitting time decreased, and no statistical interactions were detected. Sensitivity analyses which excluded participants with a history of cancer, or who were taking medications gave similar results (data not shown).

## Discussion

In this metabolic profiling study of polar metabolites in a population-based setting, we found that several amino acids and their related metabolites were strongly associated with TPA levels. Further, some of these metabolites were also associated with daily sitting time. To our knowledge, this is the first epidemiological study to report associations between polar metabolites and physical activity in a lean, healthy-weighted Asian population (average BMI 23.6 kg/m^2^). The metabolites observed here are key metabolites in the development of insulin resistance in cellular and animal studies [37,38] and in the development of diabetes in previous epidemiological studies [15,39,40].

Among the metabolites we examined, changes in plasma metabolites related to BCAA metabolism were evident. Concentrations of isoleucine, one of the BCAAs, were higher in the lower daily active groups, consistent with earlier studies [13]. Additionally, leucine and valine were also decreased in the groups with less sitting time.

Recent studies have shown associations between higher concentrations of BCAAs and future T2D risk or insulin resistance [15,39,40]. A study which compared twin pairs with an active and an inactive co-twins found increased expression among BCAA degradation gene sets in both muscle and adipose tissues [41], while another study found that increased substrate availability of BCAAs and their derivatives might contribute to further insulin resistance [42]. The baseline characteristics in our present study did not show an association between TPA level and plasma glucose or IGT prevalence, however, this might have been due to the cross-sectional design of the study. To test our hypothesis that metabolic profiles changes prior to known indicators of the development of an insulin-resistant state, longitudinal investigation in this population is warranted. Although, the mechanisms of BCAA-induced insulin resistance still remain complex. There are reports that the dietary ingestion of BCAAs is responsible for some beneficial effects of high-protein diets [43], and a population-based study, including Asian populations, showed that a higher dietary BCAA intake was associated with a lower prevalence of overweight [44]. Allowing for this, our results provide tantalizing evidence that being more active reduces the risk of metabolic diseases.

In our study, 4-methyl-2-oxopentanoate and 2-oxoisopentanoate, branched-chain alpha-keto acids (BCKAs) derived from leucine and valine, respectively, showed similar trends to BCAAs. BCAA catabolism is known to be activated during exercise in human muscles, due to the increase in energy expenditure [45,46]. Furthermore, plasma BCKA level is reported to be a predictive biomarker for T2D and impaired fasting glucose [47]. BCAAs are first converted to BCKAs in mitochondria, which are in turn used in the production of acylcarnitines [47]. This activation of BCAA catabolism supports our finding that BCKA concentration levels, as well as BCAAs, may reflect the habitual physical activity status.

Concentrations of alanine and proline, which are known as glycogenic amino acids, were higher in our inactive groups. In the fasting state, plasma glucose level is mainly maintained by glycogenolysis (glycogen breakdown) and glycogenesis (synthesis of glucose from glucogenic amino acids). Alanine and proline are involved in glycogenesis through pathways of the glucose-alanine cycle (Cahill cycle) [48] and tricarboxylic acid cycle, respectively. However, in the presence of the insulin-resistant state, which can be considered present in people with lower physical activity and a consequent lower muscle mass [49], gluconeogenesis is uninhibited [50]. This can in turn lead to the unbalanced metabolism of glycogenic amino acids. These findings help illustrate the contribution of physical activity to glucose homeostasis.

This study was carefully designed with regard to both its epidemiological aspects and the analytical aspect of metabolomics. Among its strengths, the study was conducted in a large sample under overnight fasting conditions, which allowed us to minimize metabolic variations in relation to diet and other common lifestyle factors. Metabolome analysis was conducted under a strict measurement protocol which helped reduce measurement variation in a large epidemiological setting. Moreover, the results remained unchanged after adjustment for potential confounders, including BMI, smoking, alcohol, and energy intake, and after replication analysis, and are coherent with previous epidemiological studies [13,16].

Several limitations of our study warrant mention. Because exposure measurements were based on self-reported data, we considered it inappropriate to use TPA as a continuous variable, and instead divided subjects into quartiles and analyzed using TPA level categories. Second, CE-MS method provides high-resolution, quantitative metabolome analysis for polar metabolites, while being unsuitable for non-polar metabolites including most of lipid metabolites. Relations between physical activity status and several lipids have been reported in other studies [13,16], and further profiling on non-polar metabolites in this population will be valuable. Third, given recent reports of sex differences in metabolic profiles [17,51] and the need for single-sex studies [52], we limited subjects to males. Thus, further research into the differences between sexes in metabolite concentrations is warranted. Fourth, the cross-sectional nature of our study limits any interpretation of whether a change in long-term habits to a more physically active and less sedentary lifestyle will result in a decrease in these metabolite concentrations in plasma. Clarifying this question will require both a follow-up study in this population as well as an additional study of lifestyle modification.

In conclusion, the present study demonstrates that physical activity, in terms of both TPA and sedentary behavior, is associated with polar plasma metabolic profiles as quantified by metabolomics, especially for BCAAs and BCKAs, alanine and proline. These metabolites might play a key role in the beneficial effects of increasing physical activity in reducing non-communicable diseases.

## Supporting Information

S1 Fig Flow diagram of included and excluded participants. (TIF)

S2 Fig Cross-classified multivariable-adjusted mean concentrations of metabolites associated with TPA and sedentary behavior levels. Multivariable-adjusted (age, BMI, smoking, alcohol, energy intake) mean concentrations were calculated by groups cross-classified by TPA level and sitting time. P-values for interactions between TPA and sitting time levels were tested. Participant numbers by TPA level (Q1, Q2, Q3, Q4) were n = 128, 138, 187, 192 among the short sitting time (< 3h) groups, 127, 139, 103, 100 among the medium sitting time (3- <7 h) groups, and 43, 23, 7, 6 among the long sitting time (≥7 h) groups, respectively.
(TIF)

S1 Table Characteristics of the replication population. (XLSX)

S2 Table Associations between TPA and metabolite measurements. (XLSX)

S3 Table Associations between sedentary behavior level and metabolite measurements. (XLSX)

S4 Table Summary of metabolite concentrations of the original and replication population. (XLSX)
