# Supplementary figures and images for: Metabolic Profiling of Total Physical Activity and Sedentary Behavior in Community-Dwelling Men
Source: PLoS One. 2016 Oct 14;11(10):e0164877. doi: 10.1371/journal.pone.0164877 (PMC5065216; doi:10.1371/journal.pone.0164877)

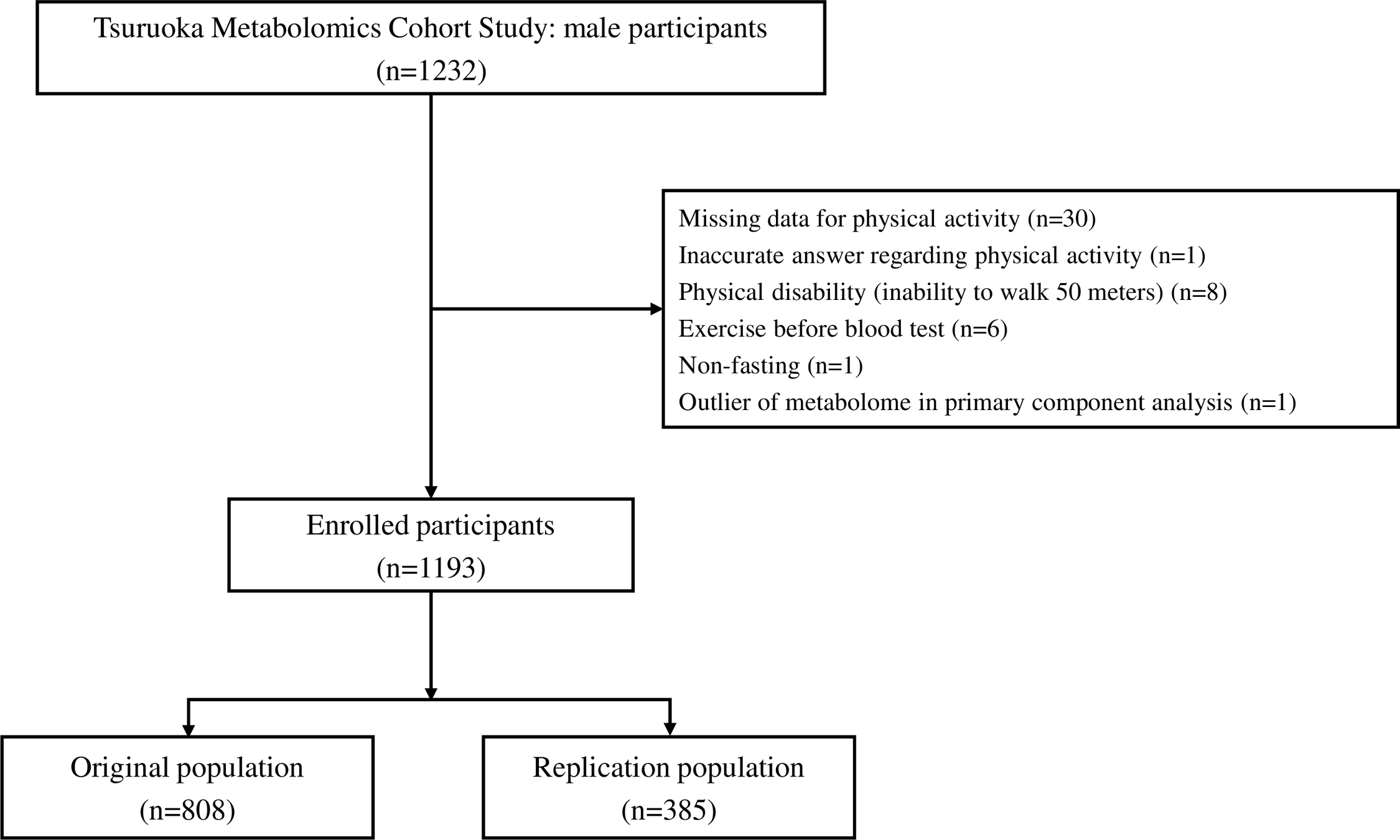

Supplement: S1 Fig — (TIF) [file pone.0164877.s001.tif]

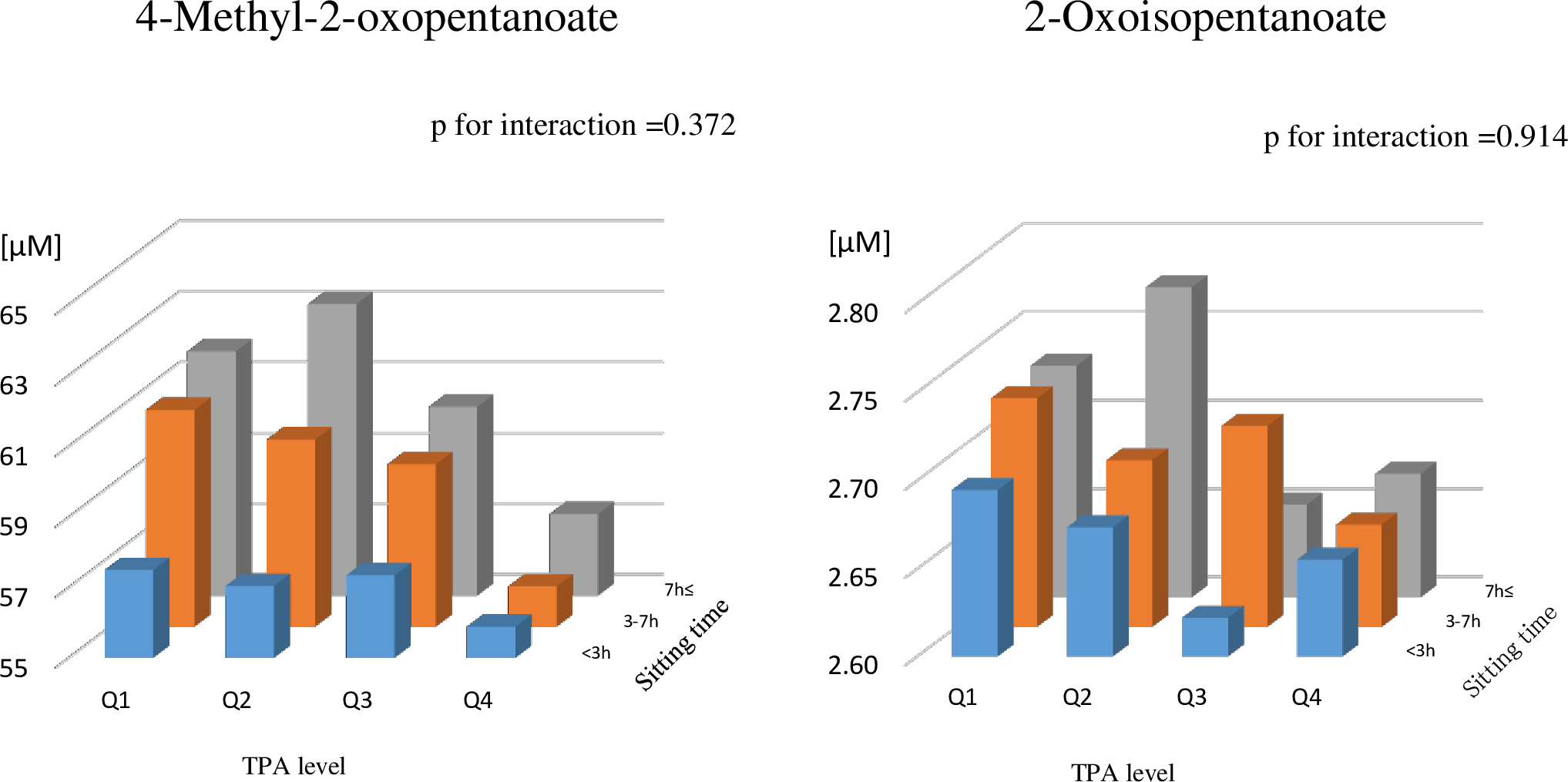

Supplement: S2 Fig — Multivariable-adjusted (age, BMI, smoking, alcohol, energy intake) mean concentrations were calculated by groups cross-classified by TPA level and sitting time. P-values for interactions between TPA and sitting time levels were tested. Participant numbers by TPA level (Q1, Q2, Q3, Q4) were n = 128, 138, 187, 192 among the short sitting time (< 3h) groups, 127, 139, 103, 100 among the medium sitting time (3- <7 h) groups, and 43, 23, 7, 6 among the long sitting time (≥7 h) groups, respectively. (TIF) [file pone.0164877.s002.tif]
